# A Case of a Rare Vascular Phenomenon: Superior Vena Cava Aneurysm

**DOI:** 10.7759/cureus.96316

**Published:** 2025-11-07

**Authors:** Hugh Jacobs, Jeremy Chan, Saifullah Mohamed

**Affiliations:** 1 Cardiac Surgery, Bristol Heart Institute, Bristol, GBR; 2 Cardiac Surgery, Royal Sussex County Hospital, Brighton, GBR

**Keywords:** aneurysm, asymptomatic, conservative management, fusiform, superior vena cava

## Abstract

Superior vena cava (SVC) aneurysms are exceedingly rare vascular abnormalities with only limited cases reported in the literature, and their natural history and optimal management remain poorly defined, particularly in asymptomatic patients. We present the case of a 75-year-old male with an incidental finding of a fusiform SVC aneurysm on computed tomography (CT). In the absence of symptoms, he was managed conservatively with anticoagulation to minimize thromboembolic risk and serial CT follow-up. Given the rarity of these lesions and the lack of standardized management guidelines, decisions regarding intervention must carefully weigh the risks of rupture and thromboembolic events against the morbidity associated with surgical repair. In this case, close monitoring with anticoagulation proved to be an appropriate strategy, emphasizing the need for individualized, risk-based management. This case highlights the importance of vigilance and long-term surveillance in patients with incidentally discovered SVC aneurysms, with conservative management and serial imaging serving as a safe option for asymptomatic individuals with stable aneurysm dimensions.

## Introduction

Superior vena cava (SVC) aneurysms are exceedingly rare vascular abnormalities, with fewer than 50 cases reported in the literature [1-4]. First reported by Abbott OA in 1950 [1], these lesions are often discovered incidentally on cross-sectional imaging, but may occasionally present with complications such as thrombosis, pulmonary embolism, or, in rare instances, rupture [3,5-6]. Because of their scarcity, the true prevalence, natural history, and optimal management strategies remain poorly understood.

Morphologically, SVC aneurysms are generally classified into two types based on their shape: fusiform, which are evenly dilated along the vein, and saccular, which are balloon-like outpouchings. This distinction is important because it influences the risk of complications and treatment choices. Fusiform aneurysms typically follow an indolent course and are managed conservatively. In contrast, saccular aneurysms carry higher risks of thromboembolic events and rupture, often prompting consideration of surgical or endovascular repair [2-6]. The aetiology of these aneurysms may be congenital, due to localized weakness in the venous wall, or acquired, secondary to trauma, infection, or prior surgery [5,7,8]. Contrast-enhanced computed tomography and magnetic resonance (MR) venography are the diagnostic modalities of choice, as they provide detailed anatomic information and help differentiate true aneurysms from other mediastinal masses or venous anomalies [5,7,8].

Given the absence of standardized guidelines, management is individualized based on aneurysm morphology, size, and symptoms. Conservative observation with serial imaging and anticoagulation has been reported as safe for stable fusiform aneurysms [3,9,10], whereas intervention is generally reserved for symptomatic or enlarging lesions [5,6,11]. Here, we present a case of an incidentally discovered, asymptomatic fusiform SVC aneurysm managed successfully with conservative surveillance and anticoagulation. This case offers meaningful clinical and academic insight and has the potential to inform future practice guidelines.

## Case presentation

**Figure 1:**
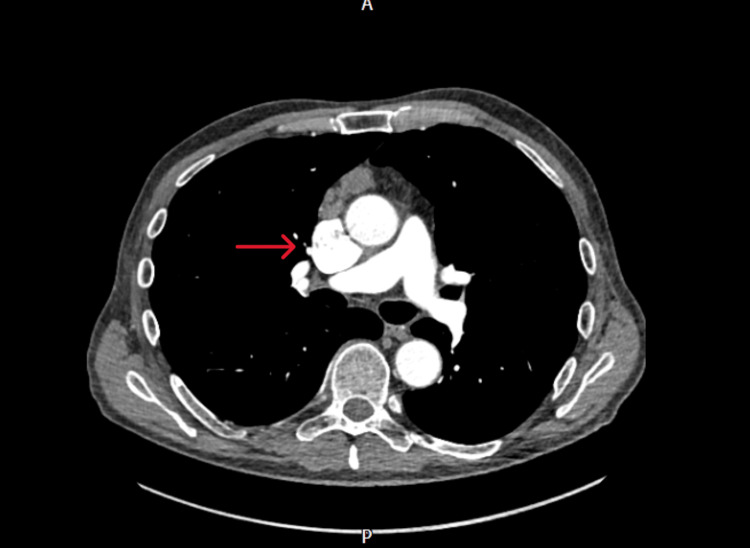
Contrast-enhanced computed tomography image 1 (axial view) The image is showing the large fusiform superior vena cava aneurysm (red arrow).

**Figure 2:**
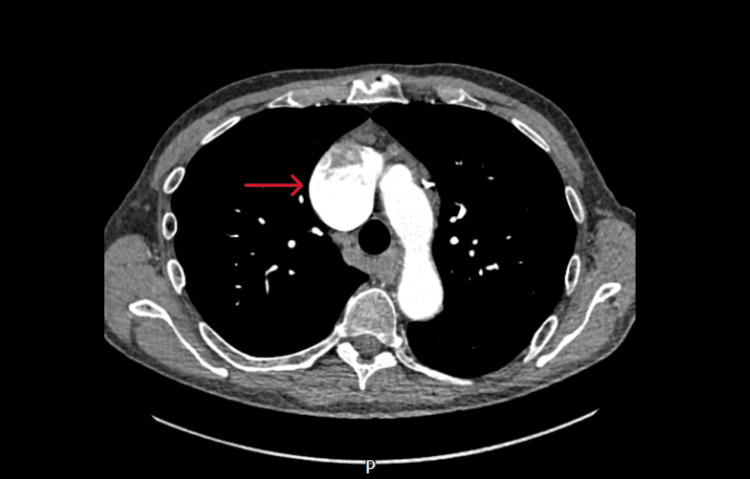
Contrast-enhanced computed tomography image 2 (axial view) The image is showing the large fusiform superior vena cava aneurysm (red arrow).

**Figure 3:**
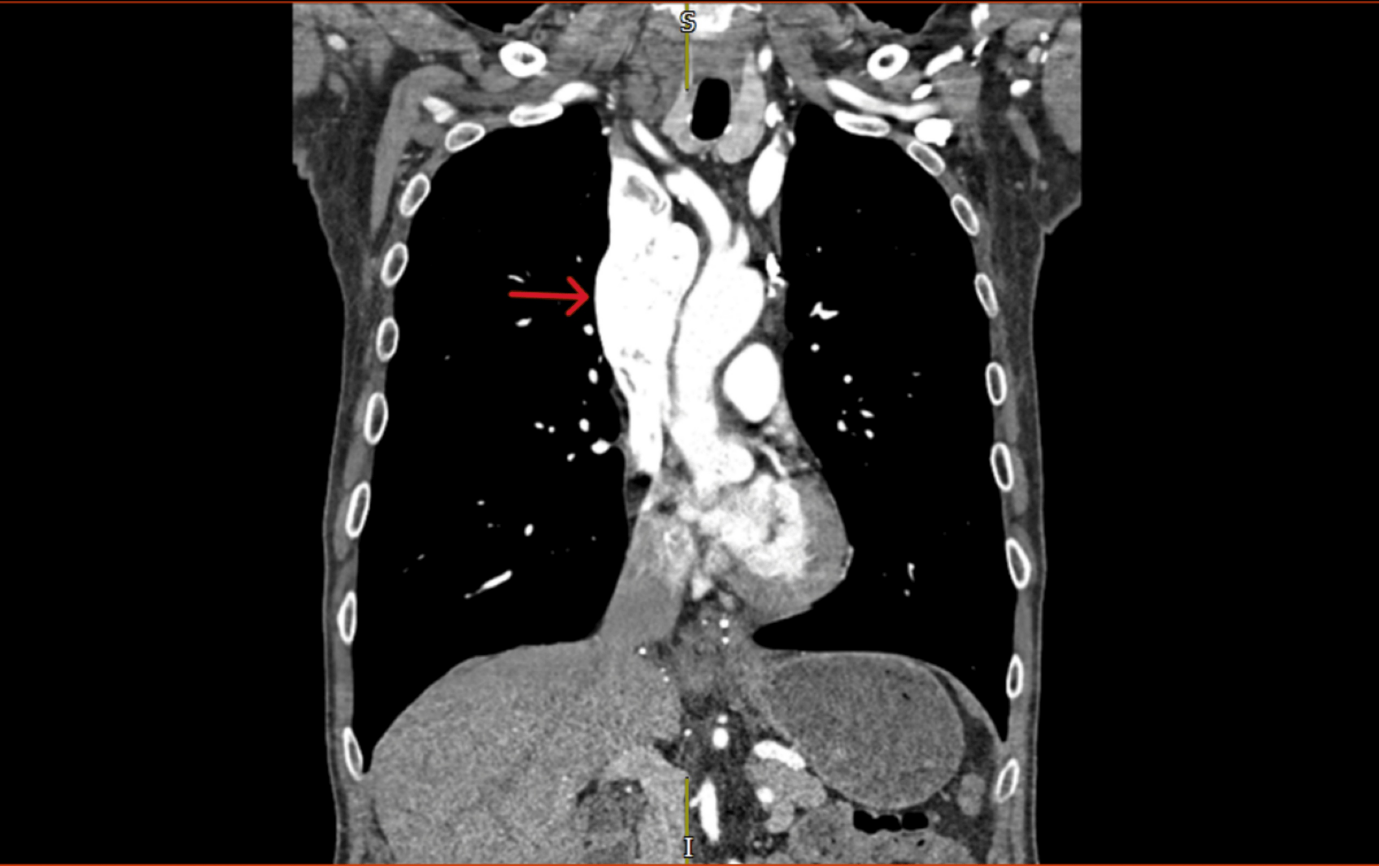
Contrast-enhanced computed tomography image (coronal view) The image is showing the large fusiform superior vena cava aneurysm (red arrow)

We report the case of a 75-year-old male referred to the cardiac surgery outpatient clinic following the incidental detection of an SVC aneurysm during routine lung cancer screening. Computed tomography (CT) imaging revealed a fusiform SVC aneurysm measuring 60 × 53 mm (Figures 1-3). Additionally, an anterior mediastinal mass with internal calcification and calcified mediastinal lymph nodes were noted, which were deemed consistent with the patient’s prior diagnosis of histoplasmosis and did not warrant further follow-up by the respiratory team. The patient was entirely asymptomatic. His past medical history was notable only for Parkinson’s disease, for which he was treated with co-beneldopa (12.5 mg/50 mg).

Given the absence of symptoms, a conservative management approach was adopted, consisting of surveillance imaging and medical therapy. The patient was commenced on a direct oral anticoagulant (DOAC), apixaban (dosage: 5 mg twice daily), to reduce the risk of thromboembolic complications. A repeat CT scan performed six months later demonstrated no change in aneurysm size, and the patient remained asymptomatic and clinically stable. Follow-up in our outpatient clinic will continue with yearly CT scans.

## Discussion

Superior vena cava (SVC) aneurysms are rare entities with no standardized management guidelines. Decisions regarding intervention must balance the risk of rupture and thromboembolic events against the morbidity of surgical repair. In this case, careful monitoring with anticoagulation proved to be an appropriate strategy, highlighting the importance of individualized, risk-based management. SVC aneurysms are rare venous dilatations, most often detected incidentally, and are classified morphologically as fusiform or saccular. Morphology correlates with risk as fusiform lesions are more commonly observed and often clinically indolent, whereas saccular aneurysms are reported to have higher rates of thrombosis, pulmonary embolism, venous obstruction, and rupture [2-4]. This risk gradient drives management toward surveillance for stable, asymptomatic fusiform aneurysms and toward intervention for complicated or enlarging saccular lesions [3-6].

Advanced cross-sectional imaging, particularly contrast-enhanced CT or MR venography, is essential for confirming venous origin, defining morphology, and evaluating for intraluminal thrombus or compressive sequelae [5,7]. Imaging also distinguishes true aneurysms from other mediastinal masses or upstream venous dilatation due to distal obstruction. Three-dimensional reconstructions can be valuable for defining the relationship to the brachiocephalic confluence and the azygos vein, especially when surgical or endovascular therapy is contemplated [8,12]. In our case, interval stability and a fusiform configuration supported a conservative management strategy with serial imaging.

Management strategies range from conservative observation to open repair and, in select cases, endovascular intervention. For conservatively managed fusiform aneurysms, many authors report safe long-term surveillance [3,9,13]. Some clinicians advocate the use of antiplatelet or anticoagulant therapy to reduce the risk of thromboembolic complications, although the evidence is limited and practice varies; therapy should therefore be individualized according to bleeding risk and imaging evidence of stasis or mural thrombus [3,9,10]. In contrast, saccular aneurysms are often managed more aggressively. Surgical intervention is recommended when there are symptoms, rapid enlargement, thrombus formation, or aneurysm size greater than 40 mm, as these features are associated with higher risks of rupture and embolic events [2,4-6].

Open surgical repair typically involves aneurysm resection with venous reconstruction using patch angioplasty or interposition grafting, often under cardiopulmonary bypass with bicaval cannulation [2,5,6]. Less invasive strategies have recently been described. For example, percutaneous balloon-protected thrombin injection was successfully used to treat a large saccular SVC aneurysm with a well-defined neck, achieving durable exclusion at 12-month follow-up and avoiding the need for a stent graft or open surgery [11]. Such endovascular options are reserved for carefully selected cases where anatomy is favorable and surgical risk is high.

In our patient, the aneurysm was fusiform, large (60 × 53 mm), and asymptomatic, with no change at six months of follow-up. These features align well with published evidence supporting conservative management of stable fusiform aneurysms [3,9,13]. The initiation of a direct oral anticoagulant was undertaken as a risk-reduction strategy; while several reports document safe surveillance without anticoagulation, this approach remains reasonable given the theoretical risk of thromboembolism and the lesion’s large size [3,9,10]. Ongoing imaging surveillance remains essential to detect any change in aneurysm morphology or size that might necessitate surgical consideration. Table 1 summarizes a few cases of SVC in adults reported in the literature.

**Table 1:** Summary of a few published cases of diagnosed superior vena cava aneurysm in adults SVC: Superior Vena Cava, PE: Pulmonary embolism, M: Male, F: Female.

Author	Year	Study design	Age/ Gender	Symptoms/ Presentation	Type of SVC aneurysm/ max diameter	Presence of thrombosis (PE, SVC or other)	Management	Outcome	Duration of follow-up
Abbott OA [1]	1950	Case report	19 M	Asymptomatic	Fusiform/70 mm	No	Surgical- wrapped with reactive cellophane	Moderate reduction in size of aneurysm. No complications or rupture.	>1 year
Gozdziuk et al. [5]	2004	Case report	59 F	Mild shortness of breath, chest pain	Saccular/90 mm	No	Surgical: resection-ligation of stalk	Good post-operative outcome.	2 years- no complications
Koga et al. [6]	2006	Case report	65 F	Asymptomatic	Fusiform/82 mm	No	Conservative: anticoagulation + follow-up imaging	-	-
Enright et al. [13]	2010	Case report	59 F	Asymptomatic	Saccular/44 mm	No	Conservative: follow-up imaging	No complications or rupture	-
Jargiello et al. [11]	2014	Case report	24 M	Asymptomatic	Saccular/Not stated	No	Percutaneous transcatheter thrombin injection	Good outcome- shrinkage to 20mm at 12 months.	12 months
Patel et al. [3]	2016	Case report	73 F	Asymptomatic	Fusiform/59 mm	No	Conservative: follow-up imaging	No complications or rupture.	6 years- no complications
Sharma et al. [9]	2017	Case report	42 F	Signs of lower respiratory tract infection.	Fusiform/55 mm	No	Conservative: follow-up imaging	No complications or rupture.	Not stated.
Nakajima et al. [8]	2019	Case report	57 F	Asymptomatic	Fusiform/55 mm	No	Conservative: follow-up imaging	No complications or rupture.	-
Jacobson et al. [2]	2019	Case report	36 F	Shortness of breath and chest wall discomfort	Saccular/122 mm	Yes (SVC thrombus and PE)	Surgical resection + anticoagulation (Apixaban)	Good post-operative outcome.	-
Morales et al. [4]	2020	Case report	75 F	Chest wall discomfort	Saccular/57 mm	No	Surgical: resection of sac and reconstruction with bovine pericardial patch.	Good post-operative outcome, discharged on day 5 post-surgery.	-
Kapoor et al. [10]	2022	Case report	47 F	Shortness of breath on exertion	Fusiform/140 mm	Yes- SVC thrombus	Conservative: anticoagulation (Apixaban) + follow-up imaging	-	-
Batouty et al. [12]	2023	Case report	62 M	Atrial fibrillation	Fusiform/65 mm	No	Conservative: follow-up imaging	No complications or rupture.	-

## Conclusions

This case underscores the importance of vigilance and long-term surveillance in patients with incidentally discovered SVC aneurysms. Conservative management with anticoagulation and serial imaging may be a safe option for asymptomatic individuals with fusiform aneurysms and stable dimensions. However, as this is a single case, a longer follow-up is required to confirm the sustained safety of this approach, and the findings may not be generalizable to saccular aneurysms, which are associated with a higher risk of complications. Continued clinical vigilance and imaging surveillance remain essential, even when lesions appear stable. This case adds to the limited literature on SVC aneurysms and may help to inform future management recommendations as additional evidence emerges.
